# Supplementary material for: Biodegradation Studies of Polyhydroxybutyrate and Polyhydroxybutyrate-co-Polyhydroxyvalerate Films in Soil
Source: Int J Mol Sci. 2023 Apr 21;24(8):7638. doi: 10.3390/ijms24087638 (PMC10146786; doi:10.3390/ijms24087638)
Supplement: Supplementary file 1 [file ijms-24-07638-s001.zip › ijms-2332395-supplementary.pdf]

Article

Biodegradation Studies of Polyhydroxybutyrate and Polyhydroxybutyrate-*co*-Polyhydroxyvalerate Films in Soil

Jihyeon Kim [1,2,†], Nevin S. Gupta [1,†], Lindsey B. Bezek [1], Jacqueline Linn [1], Karteek K. Bejagam [3], Shounak Banerjee [4], Joseph H. Dumont [1], Sang Yong Nam [2], Hyun Woo Kang [5], Chi Hoon Park [5], Ghanshyam Pilania [3,6], Carl N. Iverson [1], Babetta L. Marrone [4] and Kwan-Soo Lee [1,*]

[1] Chemistry Division, Los Alamos National Laboratory, Los Alamos, NM 87545, USA
[2] Department of Materials Engineering and Convergence Technology, Gyeongsang National University, Jinju 52828, Republic of Korea
[3] Materials Science and Technology Division, Los Alamos National Laboratory, Los Alamos, NM 87545, USA
[4] Bioscience Division, Los Alamos National Laboratory, Los Alamos, NM 87545, USA
[5] Department of Energy Engineering, Future Convergence Technology Research Institute, Gyeongsang National University, Jinju 52725, Korea
[6] General Electric Global Research Center, Niskayuna, NY 12309, USA
* Correspondence: kslee@lanl.gov; Tel.: +1-(505)-667-3060
† These authors contributed equally to this work.

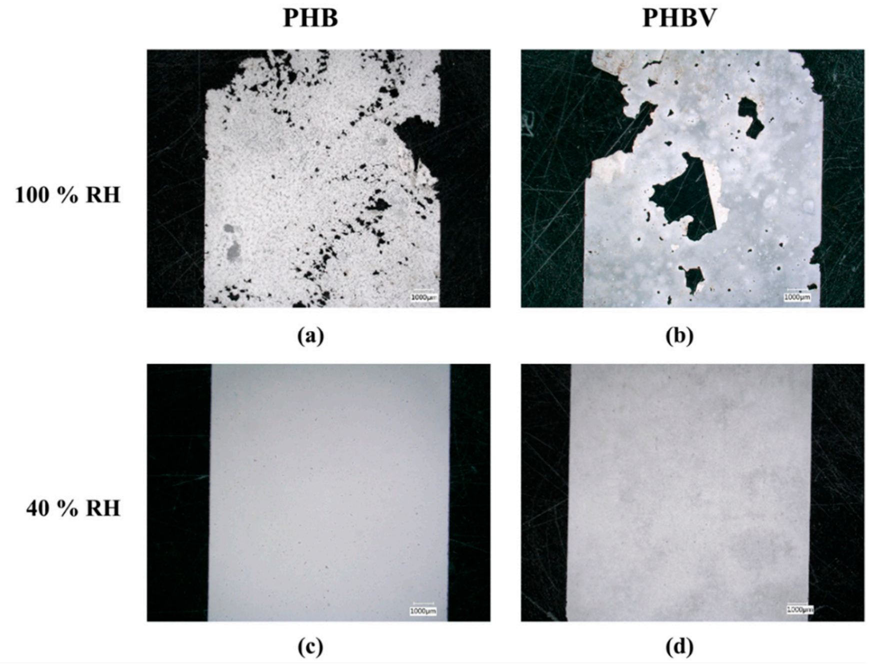

Supplemental Figure S1. The microscope images of PHB and PHBV with 7 days under 100% RH and 6 weeks under 40% RH soil conditions: (a) PHB and (b) PHBV in soil saturated with water (100% RH) for 7 days and (c) PHB and (d) PHBV in soil 40% RH for 6 weeks.

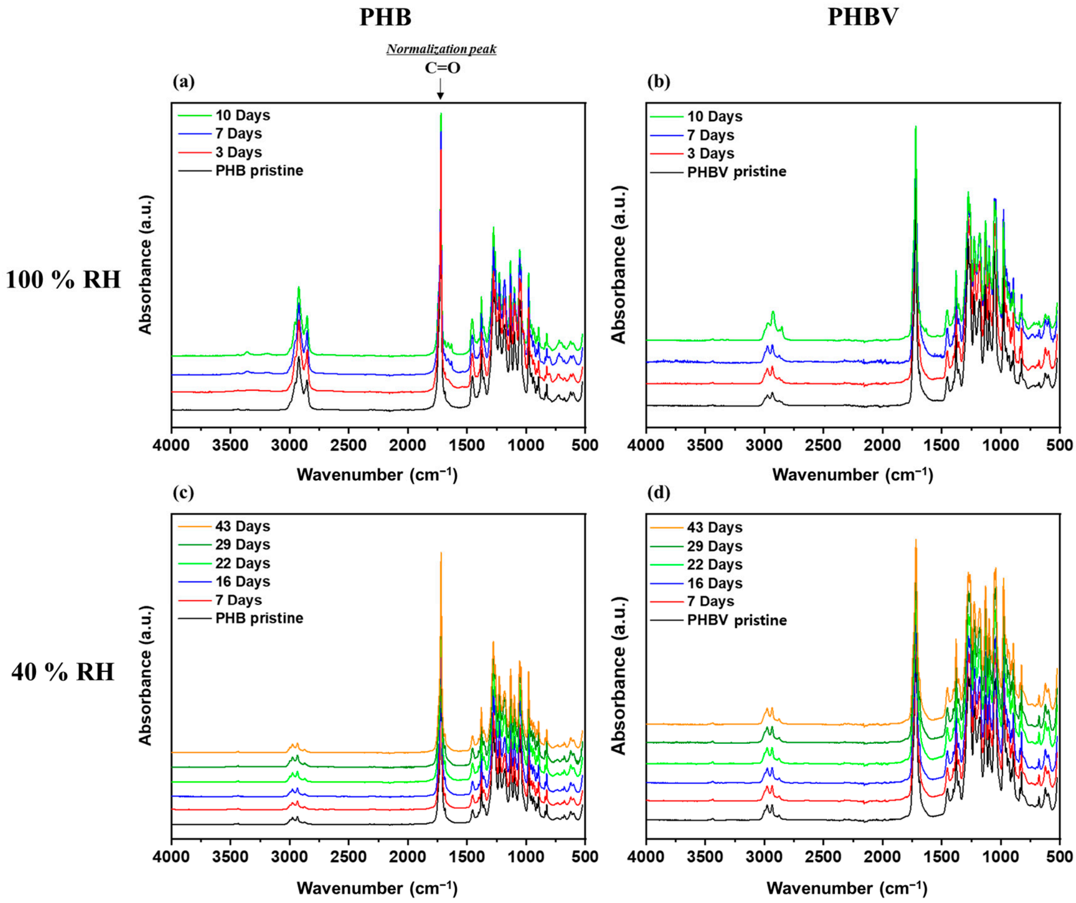

Supplemental Figure S2. FT-IR of PHB and PHBV after being aged in two different soil conditions: (a) PHB and (b) PHBV in soil saturated with water (100% RH) and (c) PHB and (d) PHBV in soil 40% RH.

Supplemental Table S1. FT-IR peak assignments of PHB and PHBV.

Position of peak (cm^{-1})	Chemical group
3550-3200	O-H stretching
3020-2840	C-H stretching
1720	C=O stretching
1453	$-CH_2$ bending
1380	$-CH_3$ bending
1276-1179, 1054-1043	C-O stretching
1021	C-OH stretching

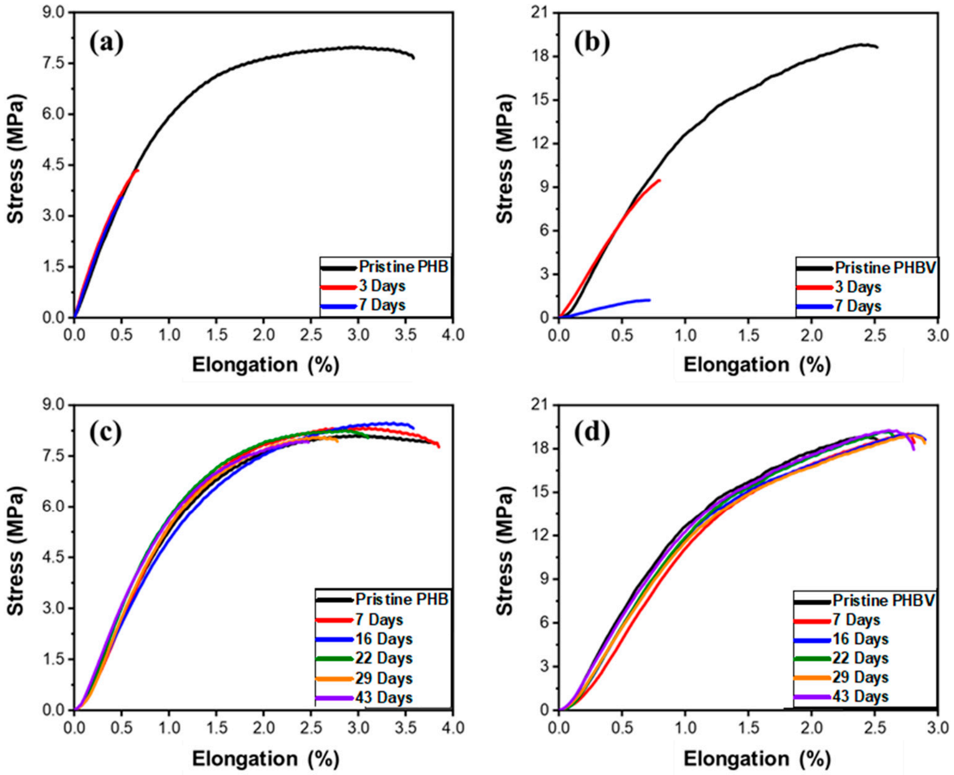

Supplemental Figure S3. Stress-strain curve of PHB and PHBV after being aged in two different soil conditions: (a) PHB and (b) PHBV in soil saturated with water (100% RH), (c) PHB and (d) PHBV in soil with 40% RH.

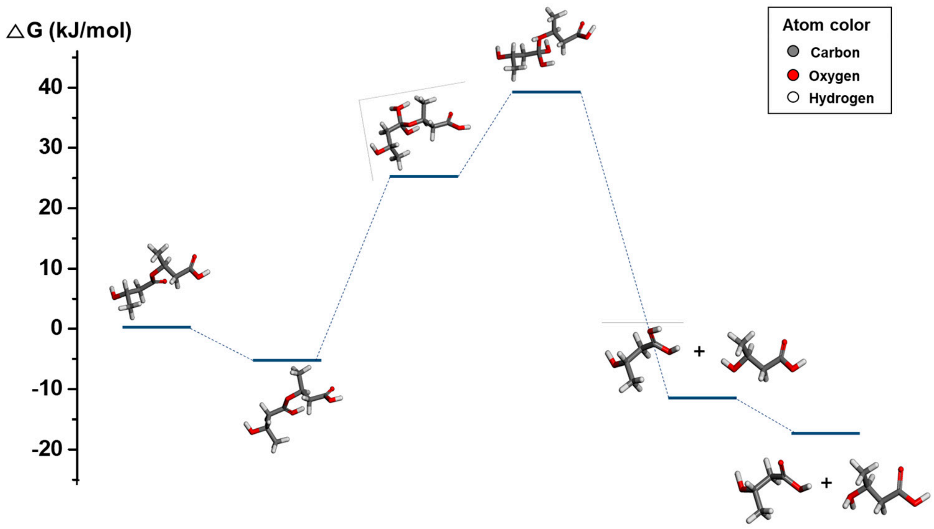

Supplemental Figure S4. The Density Functional Theory (DFT) calculation for the free energy change for PHB as it is degraded under water medium conditions.

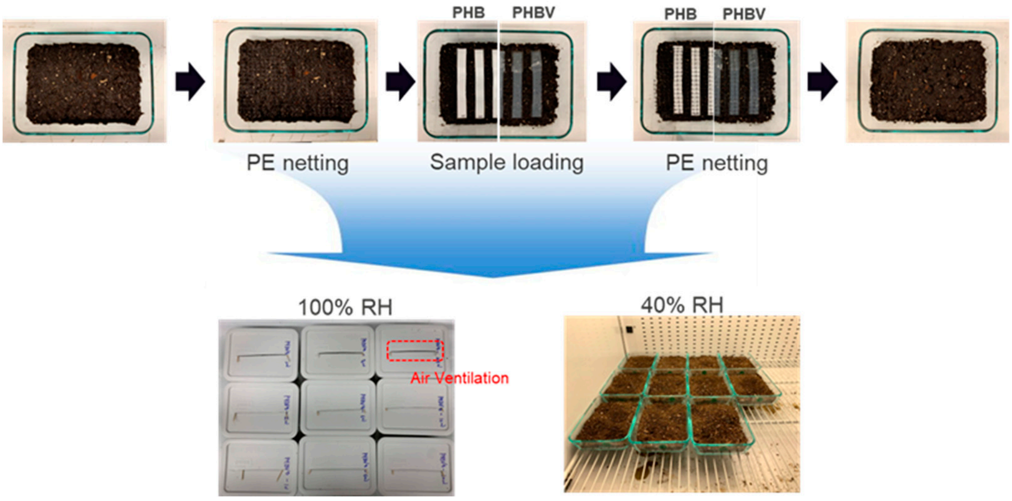

Supplemental Figure S5. The procedure used to prepare samples for the soil degradation experiment, in two different soil conditions of 100% RH and 40% RH.
